# Phospholipid-Nucleic Acid Complexation: Biomolecular Energetics of DNA-Mg^2+^-Phosphatidylcholine Ternary Complex Formation, Compaction and Relevance as Lipoplex Formulation

**Published:** 2006-12

**Authors:** Erhan Süleymanoglu

**Affiliations:** 1 *The Slovak Academy of Sciences, Institute of Experimental Physics, Department of Biophysics, Košice, Slovak Republic;*; 2 *Swammerdam Institute for Life Sciences, BioCentrum Amsterdam, University of Amsterdam, 1098 SM Amsterdam, Netherlands*

**Keywords:** polyelectrolyte phospholipid-polynucleotide nanostructures, differential scanning microcalorimetry, thermotropic phase behaviour, non-viral gene delivery

## Abstract

Thermodynamic features related to preparation and use of self-assemblies formed between multilamellar and unilamellar zwitterionic liposomes and polynucleotides with various conformation and sizes are presented. The divalent metal cation induced adsorption, aggregation and adhesion between single- and double-stranded polyribonucleotides and phosphatidylcholine vesicles was followed by differential adiabatic scanning microcalorimetry. Nucleic acid condensation and compaction mediated by Mg^2+^ was followed, with regard to interfacial interaction with unilamellar vesicles. Microcalorimetric measurements of synthetic phospholipid vesicles and poly(ribo) nucleotides and their ternary complexes with inorganic cations were used to build the thermodynamic model of their structural transitions. The increased thermal stability of the phospholipid bilayers is achieved by affecting their melting transition temperature by nucleic acid induced electrostatic charge screening. Measurements give evidence for the stabilization of polynucleotide helices upon their association with liposomes in presence of divalent metal cations. Such an induced aggregation vesicles either leads to heterogeneous multilamellar DNA-lipid arrangements, or to DNA-induced bilayer destabilization and lipid fusion. The further employment of these polyelectrolyte nanostructures as an improved formulations in therapeutic gene delivery trials, as well as in DNA chromatography is discussed.

## INTRODUCTION

The current inefficiency and toxicity issues of therapeutic gene delivery systems has been the main motivation for studying interactions of nucleic acids with phospholipids. Therapeutic gene delivery is achieved by utilizing viral or non-viral, synthetic or physical methods (1-6). Despite their well-established cell penetration properties, viral-based delivery vectors (1-5) possess immunotoxic side effects (1, 4-9). In this context, non-viral gene therapy has been proposed as a suitable alternative (4, 8-11). Non-viral nucleic acid therapy includes complexes obtained from DNA-synthetic polycations (polyplexes) (12-15), or DNA-lipid (lipoplexes) nanomixtures (2, 5, 9-11, 16). Whatever the approach is, in both polyplex and lipoplex systems, the aim is to increase the transgene expression, while improving their bioavailability and descreasing their toxicity. Therefore, the desired nucleic acid packaging becomes an objective of physical pharmacy, requiring major contributions from physicochemically oriented groups.

Achievement of stable nucleic acid-lipid formulation with controllable features is a prerequisite before starting *in vitro* transfection assays. Parameters of potential interest to be followed are phase behaviour, size and morphology, structural transitions of nucleic acids studied, induced by various condensing agents, such as various detergents with different electrostatic and hydrophobic nature, charged and neutral polymers, metal ions, as well as mixtures of cationic and anionic macromolecules, and thermodynamically stable lipid vesicles. Despite extensive research reports on nucleic acid aggregation with liposomes of various lipid composition, the colloidal factors and forces governing their complex formation remain to be understood. Additional requirements of size homogeneity, stability, ability to keep the entrapped therapeutic gene sequence in sufficient concentration and reproducible manufacturing issues render the development of such gene carriers difficult.

Following the interesting recent results of using cationic, small membrane-permeant molecules (17), as well as our own data on Mg^2+^ as bridging DNA with liposomes (18) and surfactants (19) we have focused on designs involving such small cations as rapidly moving through model cellular membranes. These could then bind nuclear DNA with high affinity.

Since understanding of energetics of DNA-lipid recognition and complexation is of major importance in this context, we have focused on the thermodynamics and structural features of lipid binding to DNA. Attempt was done for comparison of phase behaviour of phosphatidylcholine and its nucleic acid binding modes and how this affects the energy of DNA-lipid complex formation. Clarifying the affinity for DNA binding, would give us more clues on the correlation between nucleic acid compaction and transfection efficiency obtained from previous studies of DNA associations with lipid dispersions and polycations with different chain length and degree of unsaturation. In our opinion, deducing from both theoretical and experimental studies will improve the current knowledge of surface molecular interactions of these promising formulations in terms of designing improved gene delivery systems, as well as developing novel DNA chromatographic stationary phases.

## MATERIALS

Polyethylene glycol (PEG-20,000), MgCl_2_ · 2H_2_O, NaCl, synthetic polyriboadenylic acid: polyribouridilic acid (poly(A:U)_n_), 1,2-dipalmitoyl-*sn*-glycero-3-phosphatidylcholine (DPPC), SSC (1.5 × 10^-3^ mol/l Na-citrate, 1.5 × 10^-4^ mol/l NaCl, pH=7.2) reagents were purchased from Sigma (St. Louis, MO, USA) and used without further purification. 4’,6-diamidino-2-phenylindole dihydrochloride (DAPI) and FM 4-64 were a product of Molecular Probes (Eugene, OR, USA). All other reagents were of analytical grade.

## METHODS

In studies on liposomal gene delivery designs of this sort as undertaken in the current study, it is important to emphasize the item of physicochemical stability of the resulting DNA-lipid assemblies. This issue becomes especially crucial in serum environment, where major degradation problems arise. Therefore, description of general thermodynamic stability parameters of these lipoplexes is necessary before switching to real cell transfection experiments. The present study describes some basic procedures concerning the employment of differential scanning calorimetry as a generally accepted and employed procedure for building relevant physicochemical models of DNA-phospholipid recognition, binding and complexation and their relevance in designing lipoplexes with improved properties.

### Preparation of Polynucleotide Solutions and Concentration Determinations

Polynucleotides and calf thymus DNA (SIGMA) were dissolved either in SSC, Tris-HCL or HEPES buffer solutions. The concentrations of single- and double-stranded polynucleotides were determined spectrophotometrically by using the molar extinction coefficients per base pair. During all kinetic and calorimetric experiments, poly-nucleotide concentrations were 0.14 mg/l ml 10 mM buffer used/10 mM NaCl, pH=7.22. Calf thymus DNA with MW of 8.6 MDa (=13 kb) (Sigma, D4764) and specificity of 42% GC; T_m_ = 87°C, ~20 A_260_ units per mg DNA was used. The presented nucleic acid concentrations and the molar ratios are based on the average nucleotide molecular weight of 308 calculated from the known DNA composition, asemployed previously (18).

### Preparation of liposomes

Chromatographic tests for purity of the lipids were not performed, however the purity of the lipid preparation was assured from the half-widths of their main phase transitions. 1.2 mM lipid in standard SSC buffer, pH=7.2 was used in all experiments and was stored at 4°C.

Following solvent evaporation under nitrogen gas flow, the samples were left at room temperature for a couple of hours for further removal of the residual choroform by using vacuum pump. The formation of a thin layer of lipids of a 15 ml round-bottomed flask was achieved by hand-shaking and hydration in particular buffer at temperatures exceeding their main phase transition temperature. Vortexing of the lipid with the desired aqueous solution above the gel-to-liquid crystalline phase transition of the lipid (T_m_) for around 30 min resulted in multilamellar vesicles.

Unilamellar vesicles (ULV) were obtained by extrusion of multilamellar vesicle (MLV) suspension through two stacked polycarbonate filters (Nucleopore, Inc.) of 100 nm pore size at around 60°C. Repeated extrusion (10 times) through the extruder (Lipex Biomembranes, Inc., Vancouver, B. C., Canada) created homogeneous vesicle suspension. This allowed the preparation of vesicles with a mean diameter of 90 nm and a trap volume in the range of 1.5-2.0 l/mole.

### Preparation of liposome-nucleic acid mixtures

Nucleic acid-lipid mixtures were prepared 1 hour before microcalorimetric measurements by mixing of either phosphatidylcholine MLV or ULV dispersions and solvent, varying nucleic acid concentration and keeping DPPC concentration fixed. Control experiments of DNA-lipids in the absence of detergent or divalent cations, were performed in parallel. Lipid vesicles’ concentration was 0.3 mg/ml.

The preparation of phosphatidylcholine ULV-calf thymus DNA complexes, was the same as in the case of MLVs, i.e. by mixing DNA solution with aqueous DPPC dispersion in the presence of cationic surfactant or Mg^2+^. The DNA concentration used throughout all experiments was 1.8 mM based on the above mentioned assumption. A freeze-thaw protocol was followed to ensure equal distribution of solutes between lamellae and adequate hydration of the lipids. Comparison with the case of liposomal preparations without employing freeze-thaw procedure showed no difference in terms of homogeneity of the suspension. This was done by placing the sample in a cryo-tube and freezing it in liquid nitrogen for around 30 sec. The cryo-tube was subsequently removed and was plunged into warm water bath (~60°C). When the sample was thawed, the whole cycle of freeze-thawing was repeated 6 times.

### Estimation of the amount of bound DNA

For this purpose, the well-established protocol of Monnard P.-A. *et al* (1997) (20) was followed. All complexes were vizualized by fluorescence microscopy employing staining of the DNA with DAPI and staining the lipids with FM 4-64, as described (15).

### UV/VIS Spectrophotometry

The concentration of DNA was checked from ultraviolet (UV) absorption at 260 nm using the relation 1.0 absorbance unit (A)=50 μg/ml nucleic acid. The spectra of phospholipids and polynucleotides alone, or their combinations in the presence of Mg^2+^ were recorded with Shimadzu A160 double beam spectrophotometer (Schimadzu Co. Ltd., Japan) using 3 ml quartz cuvettes thermostatized within ±0.3°C by circulating water bath connected to the cuvette holder. The absorption spectra of polynucleotides and lipids were separated from each other by simultaneously performing the measurements at their corresponding wavelengths, respectively (18).

### Turbidimetric Measurements

The kinetics of aggregation between polynucleotides with zwitterionic vesicles in the presence of metal ions and surfactants was followed turbidimetrically at both below and above lipid phase transition temperature, as described above. Aggregation rate constants were calculated from the initial slopes of the turbidity (τ) as a function of time (*t*). The data obtained was evaluated over a range of metal chlorides, polynucleotides and lipid concentrations. A total of 6 turbidity measurements per each temperature mentioned was performed and the plotted results were averaged.

### Differential Scanning Calorimetry

Calorimetric measurements were performed using Privalov type high sensitivity differential adiabatic scanning microcalorimeter DASM-4 (Biopribor, Pushchino, Russian Federation) with sensitivity higher than 4 × 10^-6^ cal K^-1^ and a noise level less than 5 × 10^-7^ W. Heating runs were performed with a scan rate of 0.5 K/min. The temperature at the maximum of the excess heat capacity curve was taken as the transition temperature T_m_ and the transition width ΔT_1/2_ was determined at the transition half-height. The calorimetric enthalpy ΔH_cal_ of the transition was determined as the area under the excess heat capacity curve (18). Care was taken to ensure the reproducibility of the obtained result in terms of instrumental drift. For this, microcalorimetric measurements of melting behaviour of lipids in complex with poly(A:U)_n_ and calf thymus DNA in various lipid/DNA ratios were carried out using another instrument (SETARAM^@^ DSC microcalorimeter), equipped with Hewlett-Packard PC and with company supplied computer programme. Scanning rate of 0.5 or 1.0°C/min and scanning range between 17°C and 95°C was used throughout measurements. Amplification range was 0.250 mV with 1500 points.

### Fluorescence Microscopy

Our recent protocol for fluorescent visualization of DNA was applied (15). Briefly, model nucleic acid was visualized with Olympus BH-2 fluorescence microscope at room temperature. The microscope was equipped with 100x oil-immersion objective of the Princeton™ charge-coupled device (CCD) camera. The observed images of the DAPI-treated DNA were quantified by using computer image software Object Image™. DNA and lipid particles were seen after treatment with DAPI and FM 4-64, respectively in various concentrations until the best visualization was achieved. The quantified images were tranfered to Adobe Photoshop 5.0™ or Canvas™ and printed on a high quality dye printer.

## RESULTS AND DISCUSSION

Since, the stability of the DNA-liposome formulations is a detrimental factor for subsequent cellular studies, the first contacts between nucleic acids and lipids, namely, adhesion, aggregation of DNA onto liposomes, and energetics of cation-induced complex formation between them, are emphasized. The present work describes preliminary measurements on poly(ribo) nucleotide-zwitterionic liposome self-assembly formation as a possible alternative of the currently employed problematic cationic lipids in gene delivery, as well as for further use in nucleic acid chromatography.

The potential of complexes formed between polynucleotides and oppositely charged cosolutes for use in nucleic acid separation, purification and gene transfection is now well-established. The objective is two-fold: to employ them as a controlled pharmaceutical formulation for gene delivery trials, and for use in DNA chromatography for analyses. In both cases, the energetics of nucleic acid-phospholipid associations becomes important for deducing the resultant structures. Thus, measuring their phase behaviour, followed by their further morphological and structural characterization would provide further clues for creating thermodynamically stable lipid-DNA nanostructures.

To mimic the way of action of more effective viruses and having considered the current cytotoxicity problems of cationic lipids, our current research is focused on complexes formed between neutral liposomes and DNA, induced by inorganic and surfactant cations acting as condensing agents. The motivation for such design has come from relevant reports on stimulatory effects of Ca^2+^ and Mg^2+^ on transfection efficiency (22, 23).

**Figure 1:**
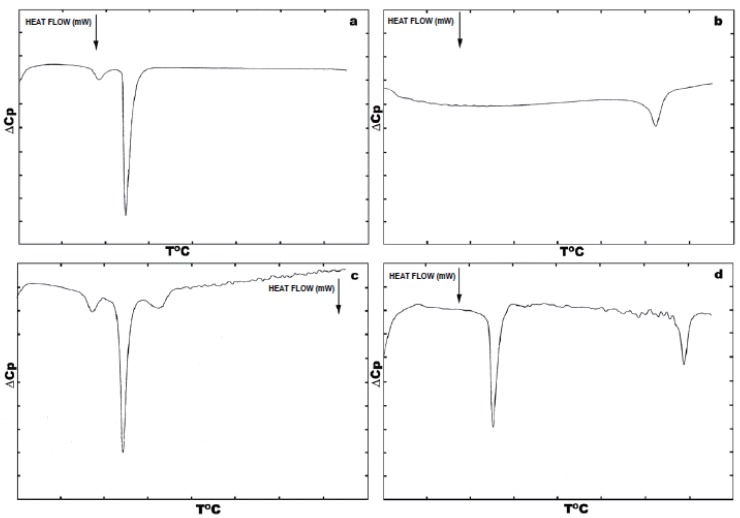
Microcalorimetric phase behaviour of DPPC multilayers in the presence of poly(ribo) nucleotides and Mg^2+^. Thermograms depict the thermal properties of ternary DNA-metal cation-lipid complexes and their components as ΔCp vs temperature plot. Heat flow is represented by the downward arrow. Concentration of synthetic biopolymers were: 1 mM of (A):(U) bp of poly(A:U) duplex, and 1 mM of DPPC (assuming average molecular mass of 770), and DNA (assuming average molecular mass of base pair of 643) in 10 mM HEPES/10 mM NaCl; pH=7.2, cell volume 1.5 ml. Differential scanning calorimetric measurements were performed using SETARAM® DSC microcalorimeter, equipped with Hewlett-Packard PC and with company supplied computer programme. (a) DPPC-MLV-Mg^2+^; (b) poly(A:U)n-Mg^2+^; (c) DPPC-MLV- Mg^2+^-poly(A:U)_n_; (d) DPPC-MLV-calf thymus DNA.

**Figure 2:**
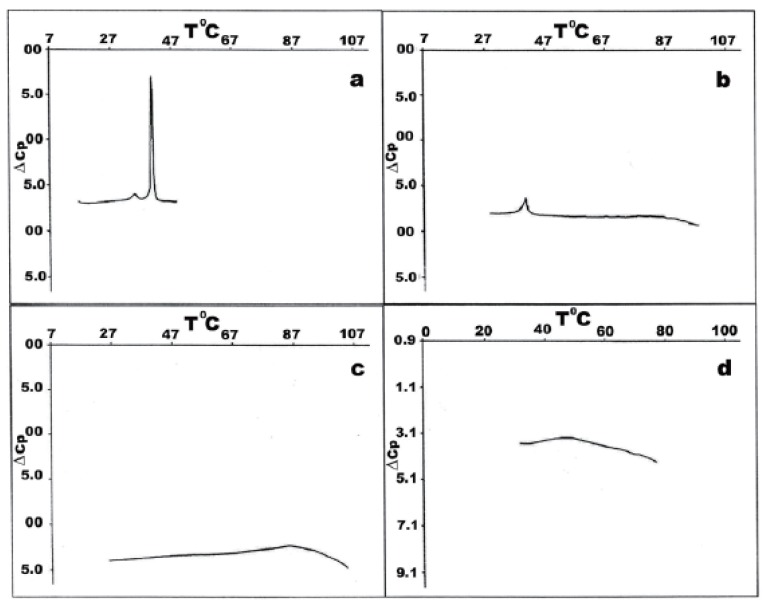
Thermotropic phase behaviour of calf thymus DNA in complex with DPPC in the presence of Mg^2+^. (a) DPPC-MLV; (b) equimolar mixture of DPPC-ULV- Mg^2+^-DNA; (c) DNA- Mg^2+^ binary complex; (d) unbound calf thymus DNA.

**Figure 3:**
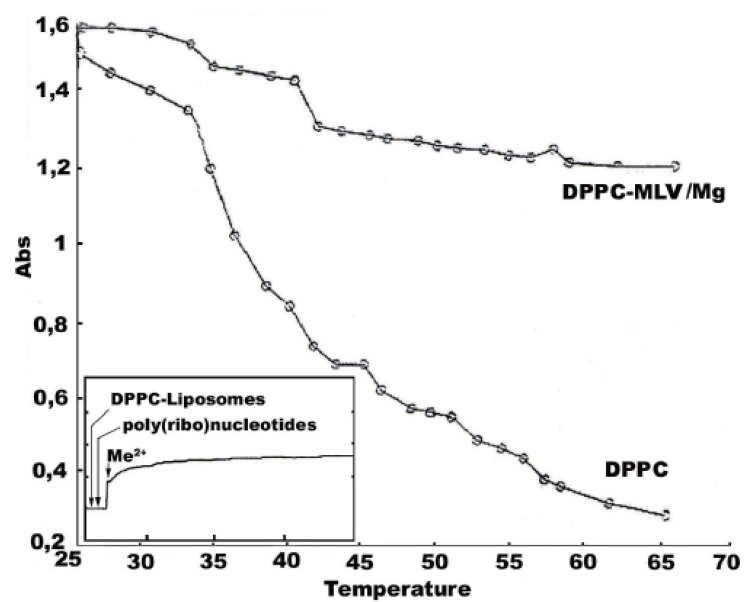
UV-melting curves of DPPC-MLV-Mg^2+^dispersions. The main phase transition tmperature of the lipids is shifted towards higher values. Thermotropic phase behaviour of lipids is presented as measurements of the change in optical density as a function of temperature. Values were recorded by stepwise increasing the temperature using 3 ml cuvettes thermostatized within ±0.3°C by circulating water bath connected to the cuvette holder, as described in Materials and Methods. Recordings were performed after maintenance of the samples in the cuvette holder for 5 min. at each teperature value. Data is presented as a mean ± S.D. of 6 measurements, and plotted using MATLAB® software. Inlet shows the aggregation and kinetics of adsorption of DPPC-MLV and ULV with poly(ribo) nucleotides or DNA in the presence of Mg^2+^ at constant temperature at 550 nm. Their initial absorbance vary with respect to each other due to their different lamelarity, curvature and homogeneity. The stock solutions of poly(ribo) nucleotides and calf thymus DNA were prepared freshly and used immediately before measurements. After zeroing the initial apparent absorption, their complexation with lipid vesicles was followed during the first 30 sec. Mg^2+^ were added to the binary mixture of ploy-nucleotides and liposomes at the final concentration of 0.5 mM and the mixture was mixed in the cuvette. The folded ternary complex is destroyed by EDTA treatment. The evaluation of their complex formation was performed, as outlined in Materials and Methods.

Fig. 1. depicts thermotropic phase transitions of DPPC multilamellar dispersions in the presence of poly(A:U)_n_ and calf thymus DNA, used here to show the effect of various length, size and conformation of nucleic acids on complexation with zwitterionic lipid, in the presence of Mg^2+^. Metal cations induce almost no any significant effects on DPPC multibilayers upon binding to them, incomparison with free lipids (Fig. 1a vs. Fig. 2a). The well-known lipid specific pre-transition (35.40°C) and main phase transition (41.68°C) temperatures (ΔH=12.51 mJ) remained unaffected (Fig. 2a). Binary mixture of poly (A:U)_n_ and Mg^2+^ results in single peak at 79.44°C (ΔH=133.81 mJ). Interestingly, the melting behaviour of calf thymus DNA (Fig. 2) shows broader transition. This represents a highly cooperative process of unwinding of the double stranded structure into two polynucleotide strands, which fold into separate chaotic globules. Fig. 1c depicts the thermotropic phase behaviour of binary mixture formed between DPPC-MLV and poly (A:U)_n_. It shows the appearance of three charactersitic peaks, corresponding to 34.36°C, 41.48°C and 50.08°C. The first two of them belong to the lipid, while the third is attributed to lipid-bound poynucleotide. Calorimetric enthalpies of these transitions are 20.78 and 138.67 mJ, respectively. On the other hand, ternary complex formed between lipid-metal cations and poly (A:U)_n_ shows the dissappearance of the pre-transition of the lipid and shifts the DPPC multilayer- bound polynucleotide phase transition to much higher temperature values of 85.88°C (ΔH=117.75 mJ). Thus, the transition depicted as DPPC-MLV- poly (A:U)_n_ → DPPC- Mg^2+^- poly (A:U)_n_ is accompanied by a change of the excess heat capacity from 2369 to 49.34 J/g. The thermograme depicts obvious effect of Mg^2+^ on duplex thermodynamic stability, as also seen from the phase behaviour of binary calf thymus DNA (Fig. 2d) as compared to that of unbound DNA (Fig. 2). The Mg^2+^-ions at the equimolar amounts with DNA increases the T_m_ value by 33.7°C, due to Mg^2+^-induced duplex stabilization. ULVs treated with the same Mg^2+^ concentration did not produce such a shift, which is normally detected spectroscopically (Fig. 3). Mg^2+^ induces the formation of substantial amount of circular DNA, suggesting that Mg^2+^ cations stabilize the interaction of polynucleotide cohesive ends, the effect being dependent on the concentration of MgCl_2_ and possibly being a sequence-specific event (26). The formed circular molecules are stabilized by Mg^2+^, but they are not covalently closed. Although, Mg^2+^ stabilizes end-to-end interactions, it is likely that a dynamic equilibrium exist between linear and circular fragments. Interestingly, the phase separation between DPPC-MLV and poly(A:U)_n_ indicates that interaction and stabilization of polyribonucleotide chain occur even in the absence of metal cation. The effect of chain length and conformation is more apparent. Specific heat capacity is higher for this polyribonucleotide in comparison with calf thymus DNA. It is still not clear whether under these experimental conditions this effect is dependent on base composition, sequence or size (18, 19), since this model nucleic acid binds to lipid surfaces in a sequence-independent manner.

**Figure 4:**
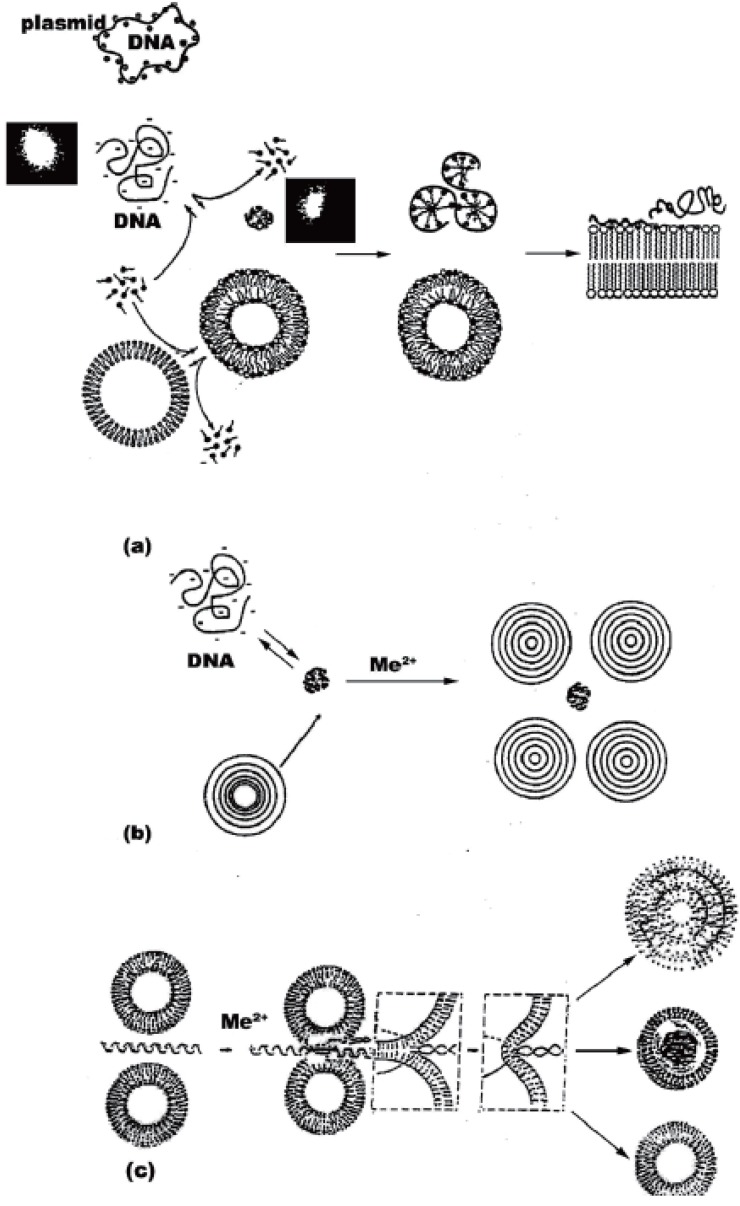
Schematic representation of formation of DNA-surfactant (a) and DNA-liposome aggregates (b-c). Plamid DNA behave differently from larger nucleic acids. (a) DNA first cover the neutral liposome with adsorbed Mg^2+^ on its surface. DNA itself leads to aggregation between two neighbouring ULVs (c). Charge neutralization brings about a local destabilization and membrane rupture and resealing of both lipid vesicle bilayers into larger structure, in which nucleic acid is sandwiched between the phospholipid bilayers. This can result in additional adhesions of other ULVs to form a multilamellar arrangements. DNA-phospholipid vesicle complexes probably fuse to a condensed and heterogeneous multilamellar structures depending on nucleic acid type and concentration. The inlets depict fluorescent images of relaxed and condensed DNA in the presence of surfactant, as described (21). Drawing is not to scale.

Fig. 2 depicts DSC heating scans of DPPC vesicles and their ternary complexes with calf thymus DNA in the presence of Mg^2+^. The first curve on the top denoted as 2-a is a calibration mark starting with a typical DPPC multilayer phase transitions, with a pre-transition temperature peak at around 36°C with ΔH_cal_ of 3.9 kJ/mol and the gel-to-liquid crystal temperature at 41.9°C. The determined parameters are in a good agreement with relevant database (LIPIDAT): http://www.lipidat.chemsitry.ohio-state.edu. The next curves show the change in phase behaviour of extruded unilamellar lipids sa a binary mixture with DNA (b) and as ternary complex with Mg^2+^ (c). Compared with pure DPPC, ternary complexes of DPPC-ULV with DNA and Mg^2+^ in equimolar ratios possess broader lipid peak with decreased maximum. The pre-transition disappears. This melting behaviour indicates that phase separation takes place between lipids, unbound nucleic acid and their complexes. Thus, after the first peak corresponding to unilamellar DPPC vesicles at 41.9°C, a second peak apears at 51.3°C for the equimolar DNA-lipid mixtures. A third minor peak corresponding to free DNA is seen at ~60°C. The observed peak distribution indicates that the fraction of liposome-free DNA is less encountered than the bound DNA in the DSC signal was detected for Mg^2+^-DPPC mixture, which was observed turbidimetrically (Fig. 4). The next curve (Fig. 2c) represents a phase behaviour of unilamellar vesicles-metal cation-DNA equimolar ternary mixture. Two peaks seen correspond to the lipid phase and to lipid-DNA agregate, respectively. The same phase behaviour and peak distribution is also observed in the case of multilamellar vesicle dispersion as binary and ternary complex with metal ions and/or polyribonucleotides (Fig. 1). The observed self-association patter of the engaged molecules is governed by surface cationization of the previously zwitterionic vesicles with their subsequent charge alteration, sensed by a conformational change in the choline group of DPPC.

Fig. 3 represents UV-melting curves of DPPC and DPPC-Mg^2+^ binary mixture. The inlet shows the aggregation and kinetics of adsorption of DPPC-MLV and ULV with poly (A:U)_n_ and calf thymus DNA in the presence of Mg^2+^, both below and above the lipid phase transition temperature, as shown by turbidity measurements. To avoid turbid solutions frequently seen during lengthy determinations, two wavelength measurements were performed, showing the same trend. The inlet depicts the aggregation pattern of the the three engaged molecular species during the first 5-10 min, since proonged assays did not result in any further significant increase in turbidity. Mg^2+^ was added at the 30th sec. Of the initial poy(ribo)nucleotide-MLV or ULV binary mixture with subsequent stirring of the cuvvette content. The physical parameter α was used as a constant to determine the degree of aggregation and surface adhesion of phospholipid vesicles with poly(ribo) nucleotides with or without metal cations, as the ratio:

α=Apolyribonucleotides−Mg2+−lipid vesicles/Apolyribonucleotides−lipid vesicles

where A is the apparent absorption. This is therefore a ratio of absorbance values measured after addition of Mg^2+^ to that before complexation with inorganic Mg^2+^. No any turbidity changes were detected in unbound poly(ribo) nucleotide samples in the absence of Mg^2+^. Interestingly, mixing them with liposomes do not lead to any turbidity change, as well (inlet of Fig. 3). Following the addition of Mg^2+^ and rapidly mixing the cuvvette content, a substantial increase of turbidity of the resulting suspension is seen, indicating the formation of large ternary aggregates. MLV showed no any turbidity increase in the presence of metal cations. The effect was observed only with ULVs. This results suggest that interlamellar repulsive forces are stronger than the exerted effects of the inorganic cations, representing a case of the effect of cation strength and valency on the structure and size of collapsed DNA. The formed toroidal DNA molecule is apparently more affected than lipid lamelarity and curvatures. This DNA aggregates ith several DPPC multibilayers, with very little tendency to penetrate at least partly to their intramolecular spaces (Fig. 4a). Polyribonucleotides and calf thymus DNA were chosen in order to test the effect of chain conformation on the complexation pattern. Thus, the observed aggregation was higher for DNA than the model ribonucleic acid, i.e.: α_calf thymus DNA_<α_poly(A:U)n_. The effect is unexpected, since the extra H-bond and the resulting duplex in calf thymus DNA should have lead to the oposite result. Apparently, the effect is determined more by the concentration, size and mobility of ploy(ribo)nucleotide helices than by their conformation. The ternary complex is destryed by treatment with 10 mM EDTA. Obviously, the divalent metal cations act as salt bridges between phosphate moieties of lipids and poly(ribo)nucleotides. Hence, the presented turbidimetric data (Fig. 3) showed that the interaction of nucleic acids from both conformations used with lipid vesicles in the presence of metal cations is characterized by increased turbidity, resulting from the liposome aggregations and fusions. Thus, increase in turbidity at lower temperatures at ~38°C and ~44°C is due to lipid fusions (18).

The UV-melting profiles were undertaken trying to depict ion induced phase transitions, which could not be detected microcalorimetrically (Fig. 1 and Fig. 2). On the contrary, temperature determined turbidity measurements indicated this effect more clearly. Apparently, divalent metal cation-lipid structures are formed, which are not detected by DSC alone. The observed discrepancy indicates the ned for coupling the calorimetric free energy estimates with one or more spectroscopic techniques. Since addition of EDTA decreased the aggregation parameter of ternary mixtures in both MLV and ULV cases, obviously metal cation affects the structure of the water bathing the lipid aggregates. This property is a function of the buffer and the ionic strength conditions used. Thus, hydrophobic and electrostatic interactions become crucial, since divalent metal cation added alters the structure of the surface water. Such a bound water exerts effects on the hydrophobic term of the free energy of the lipid aggregation and hence on lipid phase transitions, as well. When conditions of limited hydration are employed, such inorganic cations influence osmotically the lipid aggregates, by competing with them for water of hydration. Therefore, the effective number of water molecules available for amphiphile hydration is reduced. Thus, the osmotically driven lipid dehydration affects the lipids by increasing their phase transition temperature. In comparison with this indirect effect of Mg^2+^ on water structure and their distributions around the polyelectrolytes engaged in self-assembly formation, binding of these ions to charged phospholipids act by inducing lipid crystallizations, which raises the lipid T_m_.

It is worth relating the biophysical data on complexes of DNA and neutral lipid vesicles described to their possible structures formed. Apparently, structures similar to cationic-DNA complexes are likely to occur (33). Lipid-DNA arrangements are probably formed as overlaying layers of DNA adsorbed onto lipid bilayers, following charge neutralization, governed by the adsorbed cations (Mg^2+^) on the surface of the cationic lipids. The formation of alternating lipid-DNA assemblies is due to arrangements of DNA as parallel condensates between the lipid bilayers. Similar structure is reported for more biologicaly significant virus-membrane arrangements (34). This is expected, due to 3-D correlation forces between the DNA-covered lipid layers, after nucleic acid-induced formation of MLVs from ULVs. Based on our own polyelectrolyte data, as well as on literature reports Fig. 4 represents a proposal of the probable structures of ternary structures formed between neutral liposomes and DNA-Mg^2+^ complexes. The proposed structures of our recent unpublished work on *N*-alkyl-*N,N,N*-trimetylammonium (C_12_TMA) complexes with liposomes and DNA is also included for comparative purposes. The interfacial properties of the surfactant are compared with those of Mg^2+^ with regard to inducing nucleic acid compaction prior to their subsequent adsorption onto vesicle surface, as well as vesicle aggregation, adhesion, fusion and deformation. This surfactant was selected due to its interesting but yet insufficiently studied surface effects as membrane destabilizing and lipid bilayer penetrating agent. Provided its interactions with model membranes are characterized in more depth, a synthesis of novel quaternary bisammoniu compounds and further studies on their effects on cell surfaces with biomedical benefit would be stimulated.

The initially relaxed DNA in solution is complexed by C_12_TMA molecules, adsorbing on the duplex surface forming a micelle-like domains. The process of DNA compaction is shown as inlets representing fluorescent microscopy images. The surfactants also tend to form swollen mixed bilayer by partitioning in lipids. This can give rise to humpbacked vesicles with surfactant at high curvature regions. Afterwards, DNA unfolds and adsorbs on the surface of C_12_TMA-DPPC vesicles, as also suggested by a fluorescent study on zwitterionic lipids, employing cationic surfactant (35). The hydrophobically driven binding of surfactant-DNA complexes to the vesicle surface results in opening of the micelle-like domains and partitioning of C_12_TMA cations in the lipid layers. Hence, cationic surfactants apparently form fully relaxed DNA, fully bound to plasma membranes and resulting in a difficult to internalize in a cytoplasm structure by endocytosis. The drawn structures are highly dependent on size and conformation of nucleic acids. Therefore, deducing such arrangements for plasmid DNA and higher order nucleic acids should be done with precaution. Unilamellar phosphatidylcholine vesicles, on the other hand, interact with DNA by a mechanism of helix-induced liposome aggregation and subsequent lipid fusion (Fig. 4c). A lipid vesicle with higher curvature could be formed, which is preferred for further increasing of the gene transfection efficiency. Depending on particular experimental conditions, as well as on the nature of nucleic acids and lipids employed, a MLV liposome-DNA condensates are form with high probability (Fig. 4b). In contrast, stable monodisperce complexes are formed after compaction of DNA with the surfactant, followed by the addition of vesicles. Surfactants bind to DNA in a cooperative manner and increased number of nucleic acid-bound C_12_TMA leads to rise in sizes of the resulting DNA-surfactant complexes due to their aggregation. The formation of these bundles is governed by both electrostatic and hydrophobic interactions of the surfactant chains, the reaction being mediated by condensed counterions, steric hindrance or by intrinsic chain flexibility.

Mg^2+^ is tested as a compaction agent in the presence of phospholipid vesicle suspensions, in the light of its role in numerous cellular events. Its high intracellular concentrarions, as well as its well-known property of phosphate group transfer (39) makes it preferred natural divalent inorganic cation in comparison with commonly used synthetic polymers, which offer system versatility and large selection of polymer species, but are not encountered in biointerfaces. Ion transport, for instance, takes place by metal binding to cell membranes (40). Neutral phospholipids are interesting research subject not only in terms of the abovementioned preference of liposome gene delivery designers, but also concerning fundamental cell biology events. Since phosphatidylcholine moeity is a major constituent of the total phospholipid bilayer content, it is useful to study its interactions with various metals. Hence, the suggested phosphatidylcholine-Mg^2+^ binary mixture would then give further data on biological implications of metal ion control of cell membrane fluidity.

The process is hydrophobically and electrostatically controlled, since liposomes aggregate and fuse in the presence of oppositely charged particles (37). In the currently presented system, zwitterionic liposomes fuse in the presence of Mg^2+^. Hence, polyanions like DNA play an active role in adhesion, aggregation and fusion, by bridging two liposomes in close contact, with surface adsorbed metal cation-induced fusion. Since, a variety of possibilities exist regarding the structure of liposome-DNA formulations, it seems that, besides contributions due to charge neutralizations or relative lipid/DNA ratios, the absolute concentrations of the engaged system components play an additive role in thermodynamically preferred lipoplex structure formation.

The mechanisms underlying the delivery of therapeutic genes using lipid-base gene carriers is still controversial. Besides all other factors, the conformation of nucleic acids appears to play an essential role. In order to approach to certain extent one of the possible mechanisms, the involvement of induced conformational changes in the resulting phases and influence on lipid-DNA structure formation with their further relevance for transfection efficacy was studied. The physicochemical approach presented here, was performed to test the ability of single- and double stranded polynucleotide chains to promote membrane fluidity changes, vesicle surface destabilization, changed sensitivity to ions and to deduce how these features can be related to nucleic acid-Mg^2+^-mediated translocation of DNA through biomembranes. Given the structural information available from literature and based on our own data, a model for the membrane translocation can be proposed, based on the emerging relationships between transfecting cationized phospholipid and cellular anionic lipid phases.

**Figure 5:**
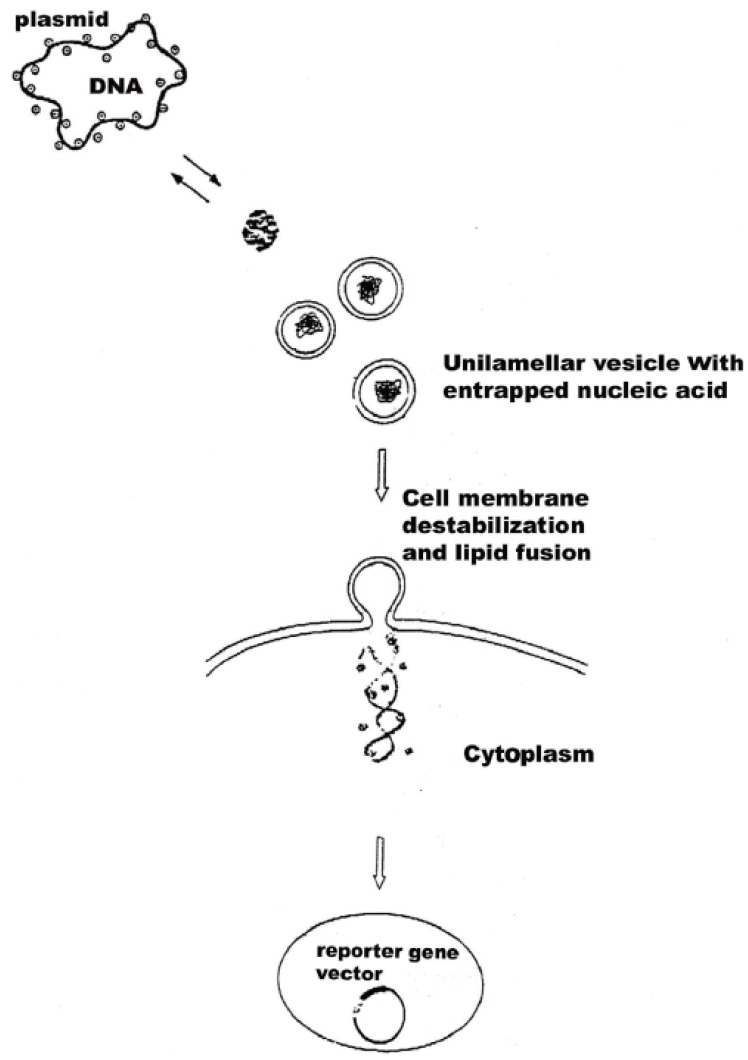
Proposed mechanism of Mg^2+^-induced internalization of neutral liposome-DNA formulations. Drawing is not to scale.

The possible mechanisms proposed for cellular uptake of DNA-liposome complexes (Fig. 5) can be summarized as the following. The first step is the formation of the nucleic acid-liposome (lipoplex) complex in either MLV or ULV form and its approaching of the target cell surface via surface forces. DNA first dissociates from lipoplexes due to neutralization by anionic lipids of the target cell surface through fusion between them. As a result, nucleic acid expands, as seen by fluorescence microscopy in a difficult to measure rates. The effect is reversible and highly depends on lipid phases. Thus, the measurable parameter such as hydration, temperature, pressure, surface tension, etc can be used to predict the particular vesicle curvature. Formation of non-lamellar phases is highly probable with further effects on transfection. The rate of fusion and DNA release is governed by the obtained higher negative lipid curvature. It is not clear at this stage whether both interfacial electrostatics and hydrophobicity are engaged in this process. Despite the previous data supporting the role of both of them, recent results from confocal imaging and fluorescence correlation spectroscopy study indicates that at least for single-stranded oligodeoxynucleotides their transfer across membranes of giant vesicles used to mimic the cell surface is similar for both negatively charged and neutral lipids and that the transfection efficiency of the lipid-DNA complexes is independent of their charge density (38). Apparently, the lipid composition of the cell membrane is more important in this type of recognition. Subsequently, the uptake of the lipoplex occurs at the aqueous membrane surface at the cellular surface, during which unfolding of the hydrophobic core of the nucleic acid is probable. Third step is composed of insertion and translocation of the polynucleotide chain into the membraneous phase, governed by lipid-lipid, lipid-protein and DNA-integral membrane protein associations. In this respect, the mechanism differs from pore formation route of cell-penetrating peptides (37, 38). The exact number of forces engaged in this structure remains to be studied. The last step corresponds to the cellular internalization of the DNA into the cytoplasm through diffusion, which is the next hurdle as it is a poor solvent reservoir for polynucleotides.

The proposal (Fig. 5) is in accordance with generally accepted principles of action of lipoplexes (1-11). DNA condensation, compaction and complexation with cations, endocytosis, and nuclear trafficking. Polyanionic DNA is apparently condensed with cationic transfection reagents, such as surfactants, or complexed with cationic or neutral liposomes, as described (Fig. 4a-c) prior to cellular targeting. These are taken up by cells mostly through endocytosis. Electrostatics governing the recognition of anionic lipids at cell surface is difficult to control. In addition, Mg^2+^ used often lead to heterogeneous size distributions of lipid-DNA assemblies. Frequently occuring MLVs (Fig. 4a) is another hurdle for uptake. Therefore recent efforts are devoted to non-electrostatic vesicle design, relying on weaker H-bond formations. The occurence of any of the four mechanisms known so far for internalization of liposomal contents in cells, namely liposome adsorption on the cell surface; adsorption of liposomes followed by selective transport of their lipophilic compounds from vesicle bilayer to plasma membrane; endocytosis of liposomes and subsequent degradation of its content and lipid fusion of vesicles with cellular surface, depends on lipid composition, charge, size, lamelarity, nucleic acid conformation and concentration, as well as on the presence of blood or serum. Due to resemblance of the viral entry route, liposome-cell fusion using fusion-inducing agents is preferrable.

Avoiding degradation of the internalized DNA by endocytic events and by cytoplasmic nucleases is a great goal. Usually, too few cells receive and express foreign DNA and even nucleic acid that has survived in cytoplasm must dissociate either before or after entry into the nucleus, for further gene transcription to occur. Despite the current desire for employment of lipid-based gene delivery tools, lack of targeting, uncertainities concerning the structures of DNA-lipid complexes and heterogeneity are problems awaiting thier solutions. In addition, if employed for systematic delivery, the DNA is subjected to blood clearance occuring as opsonization, which removes arround 90% of the hydrophobic particles in blood (1) and constitutes the major limitation of using lipid vesicles (1-11, 44, 45). Usually neutral multilamellar and unilamellar vesicles follow a slower clearance rate than negatively and positively charged MLVs. ULVs are characterized with longer residence time than MLVs (7, 9-11). The model based on data obtained with isolated macromolecules does not necessarily correspond to that existing *in vivo*. Deductions drawn from experiments with cell cultures used for studying liposome-cell interactions should be handeled with precautions, since the situation often looks different *in situ*. The events seem to be cell type-specific and show general dependence on experimental protocol, presence of serum, or other undesired complexating agents and temperature. The success of transfection assays frequently relies on particular gene reporter system employed.

To keep the model simple, we focused on relatively simple lipid phase transitions. It is clear that the mechanism proposed is oversimplified in terms of unclarified implications of this sort of delivery in intracellular trafficking and gene expression. However, data presented here, as well as that taken from the literature, give further support for generating new insights and hypotheses on non-viral gene transfer vectors. Thus, such approach helps identifying further details concerning cellular uptake of lipoplexes, DNA escape from endosomes, nucleocytoplasmic delivery and nuclear uptake. The liposome-DNA structures proposed still has to be confirmed by other methods. Transfection assays report limited efficiency with neutral lipids (36, 44). Moreover, in contrast to our suggestion, T. Stegmann and J.-Y. Legendre (1997) (32) observed that the transfection efficiency is not determined by such efficiency of membrane fusion or lipid mixing.

Animal species differences, as well as hurdles regarding *in vivo* neutral liposome-cell interactions, uncertainities of their kinetics and tissue and intra-organ distributions are issues remaining to be elucidated (44-47). However, recent reports on employment of neutral liposomes (48, 49) are encouraging to continue to study them as alternative therapeutic gene formulation.

Divalent metal cations are interesting object for use in electrostatic control of lipofection. There are several potential ways, though which metal ions can increase gene transfection efficiency. Thus, their ability to partition rapidly through cell membranes entering the nucleus may confer novel intracellular trafficking pathways on complexed DNA. Fig. 4 shows the simplified possibility of the proposed DNA-mediated liposome fusion with target cell membranes. The suggested structure of the entrapped DNA is based on Fig. 4b, originally described by Kuvitchkin and Suchomudrenko (1987) (50). The model describes the aggregation of several vesicles resulting in fusion of ULVs, induced by polynucleotide chain unwinding. Thus, the desired highly fusogenic vesicle with higher curvature is formed. Mg^2+^ used, bridge the DNA polyanion to charge reversed liposomes accelerating further their membrane destabilizing properties.

The described ability of free metal cations for both DNA and lipid binding, partitioning and permeating through cell membranes entering the nucleus (41, 42), coupled with accelerated nuclear delivery of DNA, supported by helix-binding molecules (17), is consistent with the hypothesis that divalent metal cations can confer membrane-permeant properties on complexed DNA. The exact mechanism of nucleic acid-exerted phospholipid fusion in the presence of metal cations is unknown. One possibility could be, that similarly to cationic peptides (39, 40), the electrostatic screening of the hydration shell of inorganic cations compensates for the inter-vesicle repulsive forces. Once the opposing membranes are in close contact zone, the hydrophobic and electrostatic features of metal cation form a complex between liposome and cell phospholipids followed by lipid mixing (Fig. 4). Our proposal is further supported by recent results of Sato Y, *et al*. (2003) (38) on Mg^2+^-induced DNA attachment to phospholipids.

Metal cations can govern the conformation of the transfected DNA, showing the role of nucleic acid toplogy control in gene transfection. Employment of naturally occuring divalent metal cations as oppose to other synthetic and potentially mutagenic molecules, deserves to be studies in more detail.

The concept of metal-based pharmaceuticals, undertaken here, will open new insights for studying whether these supramolecular complexes follow the similar principles of binding to cellular receptors and will further define the issue of nucleic acid receptors on cell surfaces.
